# The SPI-1-like Type III secretion system: more roles than you think

**DOI:** 10.3389/fpls.2014.00034

**Published:** 2014-02-12

**Authors:** Frank Egan, Matthieu Barret, Fergal O’Gara

**Affiliations:** ^1^BIOMERIT Research Centre, School of Microbiology, University College CorkCork, Ireland; ^2^UMR1345 Institut de Recherche en Horticulture et Semences, Institut National de la Recherche AgronomiqueBeaucouzé, France; ^3^UMR1345 Institut de Recherches en Horticulture et SemencesAgrocampus Ouest, Beaucouzé, France; ^4^Université d’Angers, UMR1345 Institut de Recherches en Horticulture et Semences, SFR4207 QUASAVBeaucouzé, France; ^5^School of Biomedical Sciences, Curtin UniversityPerth, WA, Australia

**Keywords:** SPI-1, T3SS, phytopathogens, plant, *Salmonella*, insect, amoeba

## Abstract

The type III secretion system (T3SS) is a protein delivery system which is involved in a wide spectrum of interactions, from mutualism to pathogenesis, between Gram negative bacteria and various eukaryotes, including plants, fungi, protozoa and mammals. Various phylogenetic families of the T3SS have been described, including the *Salmonella* Pathogenicity Island 1 family (SPI-1). The SPI-1 T3SS was initially associated with the virulence of enteric pathogens, but is actually found in a diverse array of bacterial species, where it can play roles in processes as different as symbiotic interactions with insects and colonization of plants. We review the multiple roles of the SPI-1 T3SS and discuss both how these discoveries are changing our perception of the SPI-1 family and what impacts this has on our understanding of the specialization of the T3SS in general.

## INTRODUCTION

Non-flagellar Type III secretion systems (NF-T3SSs) are macromolecular complexes, apparently derived from exaptation of the flagella for the delivery of bacterial effectors into eukaryotic cells (Abby and Rocha, 2012). These macromolecular complexes can be divided schematically into three parts: (i) a transmembrane export apparatus, (ii) an extracellular needle or pilus and (iii) a translocon which forms a pore in the host cell membrane (Cornelis, 2006). Additional elements, such as chaperones which can facilitate the association between effectors and the T3SS injectisome, and an ATPase which catalyzes the dissociation of the effector chaperone complex prior to secretion are also important for proper T3SS functioning (Akeda and Galan, 2005).

Multiple phylogenetic analyses based on proteins involved in the assembly of the transmembrane export apparatus have split the NF-T3SSs into seven distinct families: SPI-1 (also known as the Inv-Mxi-Spa family), SPI-2, Hrp1, Hrp2, Ysc, Rhizobiales, and Chlamydiales (Pallen et al., 2005; Troisfontaines and Cornelis, 2005; Barret et al., 2013b). From initial characterization and genomic distribution, the Ysc, Chlamydiales, SPI-1, and SPI-2 families were associated with animal–bacterial interactions while the Rhizobiales, Hrp1, and Hrp2 families were associated with plant–bacterial interactions. As well as host-range, phylogenetic groups differed in their extracellular appendages, with plant-associated families having long flexible pili, while animal pathogens have short rigid needles, which in some cases (e.g., SPI-2) can be appended by a filamentous sheath (Chakravortty et al., 2005) The core components are highly conserved between each family, which probably contributes to the phenomenon of promiscuous secretion; that is, there are multiple reports of effector secretion via non-cognate T3SS families, including effectors which are normally used during the infection of animals being heterologously expressed and secreted via phytopathogenic T3SSs and vice versa (Anderson et al., 1999; Subtil et al., 2001).

### HISTORY AND PHYLOGENETIC ANALYSIS OF SPI-1 T3SSs

**FIGURE 1:**
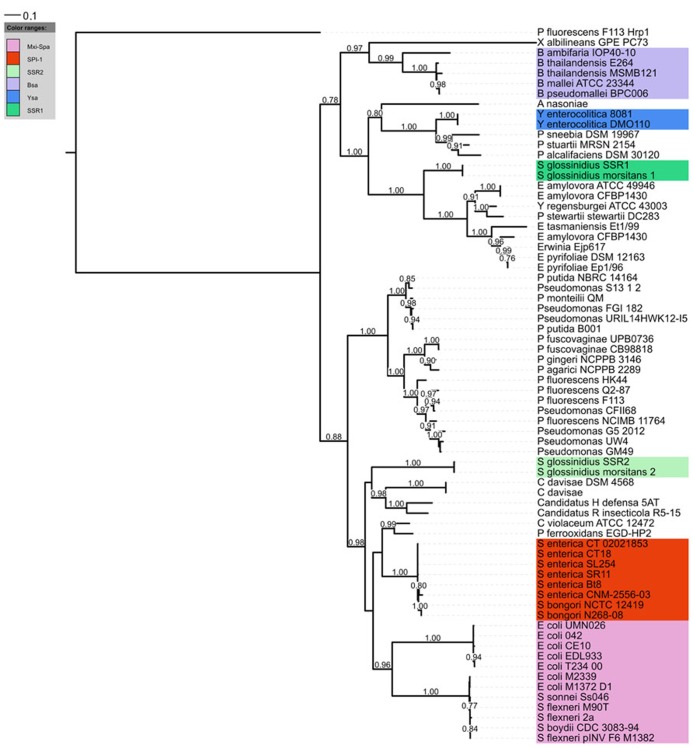
** Phylogenetic distribution of the SPI-1 T3SS**. A distance tree (maximum likelihood based on the WAG model) was calculated from InvA homologs (COG4789) of the SPI-1 family. RscV (COG4789) from the Hrp1 T3SS of *P. fluorescens *F113 was used as an outgroup. Only aLRT support values greater than 0.75 (1000 replicates) are displayed.

The *Salmonella* pathogenicity island 1 (SPI-1) is a genetic locus which is involved in invasion of non-phagocytic cells by *Salmonella* spp. (Galan and Curtiss, 1989). Molecular analyses have revealed that the vast majority of the genes located in this region encoded a T3SS (Galan, 1996). Consequently the term SPI-1 was coined for this T3SS. Homologs of SPI-1 T3SS have been identified and found to be essential virulence determinants in other mammalian pathogens such as *Chromobacterium*,* Escherichia*, *Shigella,* and *Yersinia *species (Barinaga, 1996; Miki et al., 2010), thus confirming its role in animal/human pathogenesis. However, recent reports in the literature have highlighted the presence of SPI-1 T3SSs outside of mammalian pathogenic bacteria, such as *Sodalis* (Dale et al., 2005), *Erwinia* (Triplett et al., 2006), *Xanthomonas* (Alavi et al., 2008; Marguerettaz et al., 2011), *Pantoea* (Correa et al., 2012), and *Pseudomonas *species (Barret et al., 2013a; Redondo-Nieto et al., 2013). Concurrently, the rise in genome sequences is revealing that the SPI-1 T3SS is found in a multitude of other bacterial strains (**Figure 1**).

Phylogenetic analyses have consistently split the SPI-1 family into 2 sub-clusters (Marguerettaz et al., 2011; Abby and Rocha, 2012; Barret et al., 2013b). One sub-cluster has a greater frequency of classical enteric bacteria such as *Salmonella*, *Shigella*, and *Escherichia coli*, while the other contains other bacterial strains associated with plant insect or soil environments. However, both environmental strains and mammalian pathogens can be found in each sub-cluster so it is difficult to see that this phylogenetic split has any meaningful implication for host range. Of course there may be other interesting features related with phylogeny, for instance through evolution some differences in T3SS apparatus or secretion mechanism might arise which could be particular to certain phylogenetic branches. One possible example might be that *Pseudomonas* strains possessing the SPI-1 T3SS do not have a strong homolog to the *invJ* gene which controls needle length, suggesting the needle length or regulation thereof is different in these strains.

### MULTIPLE ROLES OF SPI-1 T3SSs

**FIGURE 2:**
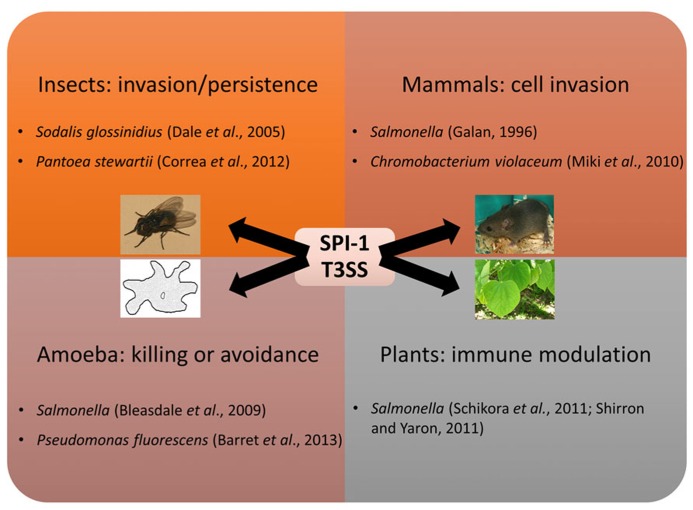
** Multiple roles of the SPI-1 T3SS**. The SPI-1 T3SS was first implicated in virulence towards mammals, but has since been shown to mediate interactions with other animals, protozoa, and plants.

As mentioned, the SPI-1 T3SS is best known for its role in mammalian pathogenesis, but it is being recognized as being important in a many different settings (**Figure 2**). The high occurrence of SPI-1 T3SS in genome sequences of insect symbionts such as *Arsenophonus nasoniae, *“*Candidatus Hamiltonella defensa*”*,* “*Candidatus Regiella insecticola*,” and *Sodalis glossinidius* could indicate that SPI-1 is often necessary for persistence in insect hosts, expanding the known host range of the SPI-1-type T3SS and demonstrating a non-pathogenic function for this T3SS family. Indeed, two SPI-1 family T3SS are found in *S. glossinidius, *one which is required for cell invasion and another “needleless” T3SS which is required for replication of this bacterium in insect cells (Dale et al., 2005). Interestingly, a recent study has also highlighted that the SPI-1 T3SS of *Pantoea stewartii* is required for persistence of this bacterium in the flea beetle, an important vector for this maize pathogen (Correa et al., 2012). Moreover, protein-coding genes involved in the assembly of the SPI-1 NF-T3SS are also abundant in arthropoda-associated microbiomes (Barret et al., 2013b).

Other SPI-1 T3SSs are involved in interactions with free-living protozoa. SPI-1 T3SS gene expression of *Salmonella* and *Pseudomonas fluorescens* is induced during contact with amoebae (Bleasdale et al., 2009; Barret et al., 2013a). In *Salmonella* a modest effect was seen on amoeba survival after *hilA*, a key regulator of the SPI-1 system, was mutated, but as non-T3SS genes are also regulated by HilA the contribution of the SPI-1 T3SS to this phenotype is not clear (Tezcan-Merdol et al., 2004). The presence of the SPI-1 system did not enable the plant-growth promoting rhizobacterium *P*. *fluorescens* F113 to survive in or kill amoeba, but instead helped the bacteria avoid amoeboid grazing. As the amoeba still consume *P. fluorescens* F113 in the absence of alternatives, it is not clear whether this is the main function of the SPI-1 system, or a useful additional function.

Finally, SPI-1 T3SS is also important during bacterial–plant interactions. For instance SPI-1 and SPI-2 T3SS deficient strains of *Salmonella* spp. are compromised for survival *in* *planta* while the SPI-1 mutant had reduced ability to repress the hypersensitive response of *Arabidopsis* (Schikora et al., 2011). In addition, the SPI-1 T3SS of *Salmonella* is also involved in suppression of the early plant immune response of *Nicotiana tabacum* (Shirron and Yaron, 2011). In contrast, the SPI-1 T3SS of *Salmonella enterica* elicits *Medicago* defense, which limits bacterial colonization of plant tissue (Iniguez et al., 2005). Plant defense responses are often categorized as either PTI or ETI, based on whether they are triggered by pathogen-associated molecular pathogens or effectors, respectively. While the structural apparatus of the T3SS might elicit the *Medicago* PTI, mutants lacking the translocator SipB reached higher numbers *in planta*, suggesting the plant’s response might be at least partially due to the presence of translocated effectors. Indeed, one effector of the SPI-2 T3SS, SseF, has been shown to trigger the hypersensitive response in *N. benthamiana* when expressed transiently or translocated by the T3SS of phytopathogen *Xanthomonas* *campestris*,**though the presence of* S. enterica *did not elicit this response (Üstün et al., 2012). While there are other examples of multi-host pathogens that infect both plants and animals, this is the only reported instance of a bacterium using the same secretion system in both processes, though *E. coli* does use its extended T3SS filament for plant attachment, and encodes several homologs to phytopathogenic effectors (Tobe et al., 2006; Shaw et al., 2008).

The SPI-1 system might also play a role in cell aggregation similar to, but independent from, biofilm formation. Indeed, when overexpressed on a vector, the SPI-1 locus induced clumping of *S. enterica* Typhimurium in media, seemingly due to an extracellular sheath composed at least partly of SPI-1 proteins (Jennings et al., 2012). Somewhat analogously the Hrp T3SS is involved in pellicle formation in *Erwinia chrysanthemi* (Yap et al., 2005). In light of the lack of evidence for SPI-1-mediated effector translocation into plant cytosol it remains a possibility that SPI-1 induced formation of cell aggregates *in planta *could shield the bacteria from plant defense receptors, though visual evidence from *Salmonella* inoculation of *Arabidopsis* has not shown large clumps of cells (Schikora et al., 2008). It is harder to envisage how cell aggregation could explain the SPI-1 dependent induction of the *Medicago* plant defense in response to infection with *S. typhimurium*,**but in these set of experiments only a translocator was mutated, so it is possible the phenotype seen was due to perception of this translocator instead of perception of translocated effectors within the plant cytosol.

### THE T3SS TRANSLOCATION PROCESS IN PLANTS AND ANIMALS

While T3SS function was initially understood within the confines of the model system(s) that are still predominantly used to characterize it, there has been a growing recognition that bacteria can utilize the same T3SS to mediate interactions with several different hosts (Preston, 2007). A role for the same T3SS with both plant and animal hosts is the ultimate demonstration of the flexibility of the T3SS, as this challenges the classical separation of NF-T3SSs from animal and plant-associated bacteria, which mainly differ in their translocators and extracellular appendages. This is presumably because of the greater challenge of delivering effectors across the additional barrier of the cell wall, which can be reinforced through callose deposition at sites of infection (Luna et al., 2011). Instead of the short (~60 nm), apparently ancestral, needle, the Hrp-T3SS has a long (~1–2 μm) flexible pilus presumed to be an adaptation to the thickness of the plant cell wall (Cornelis, 2006). The phytopathogenic translocator repertoire is more complex and variable than that of animal pathogens, which simply have three essential translocators. Though proteins involved in translocation which show homology to one particular animal translocator, SipB, can be found in some phytopathogens, this is not universal (Meyer et al., 2006). Instead, small, heat stable proteins known as harpins, which are exclusive to and possibly ubiquitous in, bacteria possessing the Hrp T3SS, have been implicated in the translocation process (Kvitko et al., 2007; Choi et al., 2013).

In light of the plant-related activities of the SPI-1 system it seems that having a long flexible pilus and harpins to facilitate delivery of type 3 effectors (T3Es) into plants is not a strict requirement, but given the strong association between the Hrp T3SS family and phytopathogens it seems likely that these factors are advantageous. How *Salmonella* could deliver effectors into the plant cell cytosol remains to be determined, though formation of elongated needles through dysregulation of needle length control has been observed *in vitro* following mutation (Kubori et al., 2000). It is important to note, however, that translocation of effectors into plant cells by *Salmonella* remains to be demonstrated.

It is interesting to note that *P.* *fluorescens* MFM1032 use a T3SS which probably belongs to the Hrp family to lyse macrophages and interfere with the growth of amoeba (Sperandio et al., 2012). Unfortunately, in the absence of a genome sequence it is not clear whether this strain possesses the plant-associated pilus and translocator repertoire.

### SPI-1 TYPE 3 EFFECTORS

Each bacterial species delivers its own unique set of T3Es into host cells and it is necessary to define their role(s) to fully understand any T3SS-dependent phenotype.

Unfortunately, from the SPI-1 family only effectors from classical mammalian pathogens have been characterized to date. As these effectors have been extensively reviewed elsewhere (McGhie et al., 2009; Parsot, 2009), the examples in the next section are not intended to be exhaustive, but instead serve to emphasize the commonalities of T3Es generally by comparing effectors secreted by the SPI-1 and Hrp families.

Although extremely diverse, described T3Es are composed of less than 40 motifs or domains which can often interfere with conserved eukaryotic cellular processes such as the mitogen activated protein kinase (MAPK) signaling pathway (Dean, 2011). Indeed, several effectors which bacteria use during infection of plants or animals have been characterized after heterologous expression in non-host yeast (Siggers and Lesser, 2008; Tripathi et al., 2010; Salomon et al., 2011). Therefore, it is possible that *Salmonella* uses a subset of the same effectors to affect both plants and animals by interfering with the same processes in both.

Many described SPI-1 effectors can be categorized as being involved in host cell invasion or host immune response modulation, and many T3Es secreted by Hrp T3SSs are also dedicated to downplaying the host immune response (Grant et al., 2006). This is especially pertinent as SPI-1 dependent phenotypes in plant models all result ultimately from differential plant immune response. SPI-1 T3Es SptP and AvrA act to inhibit the action of the MAPK-dependent immune response pathway while Hrp effector HopAO1 possibly acts downstream of the MAPK activation to suppresses plant defenses (Murli et al., 2001; Lin et al., 2003; Underwood et al., 2007; Jones et al., 2008). Hrp effector AvrptoB and SPI-1 effector SopA are ubiquitin ligases which also modulate the host immune response (Abramovitch et al., 2006; Zhang et al., 2006). SopE activates caspase-dependent immune responses in macrophages (Hoffmann et al., 2010), while caspase-like proteases also control programmed cell death in plants (Hoffmann et al., 2010; Woltering, 2010). The SptP T3E has an additional function as dephosphorylase of the mammalian ATPase valosin-containing protein (Humphreys et al., 2009), and could potentially have the same activity with the plant homolog AAA+ ATPase (Shi et al., 1995).

Other conserved targets of both plant and animal SPI-1 systems are suggested by similar localization patterns, e.g., Hrp effectors HopG1 and HopAA1-1 and SPI-1 effectors SipB and SopA are reported to be able to localize to the mitochondria (Hernandez et al., 2003; Layton et al., 2005; Munkvold et al., 2008; Block et al., 2010). Understanding these underlying commonalities we can begin to appreciate how multi-host T3SS-dependent interactions evolve as well as form hypotheses about which effectors are utilized by *Salmonella* in interactions with plants. Alternatively, it is possible that the *Salmonella* SPI-1 T3SS might translocate an entirely different, yet uncharacterized, set of proteins into plant cells.

Only recently have SPI-1 T3Es from outside mammalian pathogens been identified, and their mode of action remains to be determined. Using a conserved chaperone-binding domain (CCBD) sequence, recognized by class IB chaperones, two SPI-1 T3Es of *S.* *glossinidius* (SG0576 and SG0764) were predicted, and then confirmed to be secreted in a T3SS-dependent manner (Costa et al., 2012). This CCBD sequence could be employed to detect others SPI-1 T3Es in genome sequences possessing class IB chaperones, such as *P. fluorescens *HK44 and *Pseudomonas *sp.**GM49 (Barret et al., 2013a). Bioinformatic prediction has also been used to identify effectors. Two putative protein coding-genes (PSF113_1802 and PSF113_4041) were predicted to be potential effectors of *Pseudomonas* strains, since they possess T3 secretion signal, are only encoded in *Pseudomonas* genome sequences encoding the T3SS SPI-1 cluster. Though their translocation remains to be confirmed, their expression is induced by the SPI-1 T3SS transcriptional activator HilA in *P. fluorescens* F113 (Barret et al., 2013a). As well as using CCBD sequences to detect other SPI-1 T3Es in genome sequences, improving *in* *silico *prediction models, which are mainly based on the amino acid frequencies and biochemical properties of the effector’s N-terminal region, may result in more T3Es being found, allowing us to develop our understanding of the SPI-1 T3SS in its many diverse roles (Wang et al., 2011; Wang et al., 2013).

## CONCLUSION

Phylogenetic analyses have highlighted seven different families of T3SS and have been useful for unraveling the evolutionary history of the system, as well as for highlighting the relationship between T3SS family and (a) the specificities of its translocation process as well as (b) broader categories of target organisms. The SPI-1 system is associated with and best understood in the context of mammalian pathogenesis, but as our knowledge advances it becomes clear that this is a system with multiple functions in different hosts, perhaps especially with insects as it is particularly abundant among insect related bacteria. In light of the division between T3SS families that were found to target plant or animals in early T3SS research, it was tempting to speculate that certain T3SS families were limited to plants or non-plants but evidence is mounting that this is not the case. This raises questions about our assumptions about the specialization of the plant-related translocation process. This, and the growing appreciation of the functional relatedness of the diverse set of T3E, suggests that the various bacterial–eukaryote interactions mediated by the T3SS follow similar templates.

Though the original categorical divisions between phytopathogenic and animal pathogenic processes are being eroded, this paradigm is still useful as long as we remember that in biology, exceptions are the rule. What adaptions are needed for the SPI-1 system to interact with plant cells is an intriguing question, and when the Hrp systems are so strongly associated with plants, the prospect that no adaptions are needed is maybe more intriguing still.

## Conflict of Interest Statement

The authors declare that the research was conducted in the absence of any commercial or financial relationships that could be construed as a potential conflict of interest.
